# *The Organon* and Materia Medica Club of the Bay Cities of California

**Published:** 1897-08

**Authors:** W. E. Ledyard

**Affiliations:** B. A., M. B., M. R. C. S. Eng., Secretary


W. E. LEDYARD, B. A., M. B., M. R. C. S. Eng.,
*Secretary.*

---

### *THE ORGANON* AND MATERIA MEDICA CLUB OF THE BAY CITIES OF CALIFORNIA.

The regular semi-monthly meeting of the Club was held at the office of Dr. Selfridge, 1068 Broadway, Oakland, cor. Twelfth Street, on Friday, March 5th, 1897.

The meeting was called to order by the President, Dr. J. M. Selfridge, at 8.15 P. M.

Present: Drs. J. M. Selfridge, Geo. H. Martin, G. J. Augur, O. Swayze, C. J. Holmgren, and W. E. Ledyard.

The reading of the minutes was, by motion, omitted.

The name of Dr. Eleanor F. Martin, having been favorably passed upon by the Board of Censors and Club some months ago, she was declared by the President to be duly elected as a senior member of the Club.

*Dr. Geo. H. Martin*, as Chairman of the Board of Censors, reported favorably on the names of Drs. James W. and Florence N. Ward, 606 Sutter Street, San Francisco, for junior membership of the Club.

The ballot was unanimously in their favor, and accordingly the President declared them duly elected as junior members of the Club.

*Dr. J. M. Selfridge* read a paper entitled, "A Case from my Note-Book," *to show the efficacy of the indicated remedy in removing a fibrous uterine tumor.*

The Club then commenced the reading of Tafel's translation of Hahnemann's *Chronic Diseases.*

*Dr. J. M. Selfridge* read the preface to the fourth volume.

In discussing the preface Dr. Selfridge put the query: "Is it a fact that the human organism cannot, by the vital force unaided, bring about a cure without loss of vitality and sacrifice of animal fluids? What is vital force?"

What is vital force?

*Dr. Ledyard*—"Vital force is life, and life is spirit."

*Dr. Geo. H. Martin*—Life is the continuous adjustment of internal relations to external relations.

*Dr. J. M. Selfridge*—Life is an essence; none but Omnipotence knows what it is.

*Dr. Swayze*—If we could explain this we should have the secret of Homœopathy.

*Dr. J. M. Selfridge*—If there were no morbific forces we should have no sickness. Hahnemann also calls this principle, "spirit-like" vital force. He considers it the principle of life.

"The preface to the fifth volume" was also read by the President. A discussion followed with regard to the difference between *dynamization* and *dilution.*

*Dr. J. M. Selfridge*, referring to note to Section 230 of *The Organon,* said: "This process (dynamization) sets free the molecules and the atoms. The molecular theory of matter was unknown to Hahnemann."

The Club then adjourned to meet on the third Friday in March, at the office of Dr. Geo. H. Martin, 606 Sutter Street, San Francisco.

